# On "Non-Restraint," in the Treatment of the Insane, and the Increase of Lunatics
**Le Non-Restraint, ou de l'Abolition des Moyens Coercitifs dans le traitement de la Folie, suivi de considérations sur les causes de la Progression dans les nombre des Aliénés admis dans les Asiles.* Par M. le Dr. Morel, Medicin en Chef de l'Asile de Saint-Yon (Seine Inferieure).


**Published:** 1861-01

**Authors:** 


### ART. X.—ON "NON-RESTRAINT," AND THE INCREASE OF LUNATICS.*

IN the treatment of the insane Dr. Morel, the distinguished physician-in-chief of the lunatic asylum of Saint-Yon (Seine-Inferieure), and well known as the learned author of several important works on medical psychology, has very recently published a report of a visit which he made to England in 1858 in order to examine into the system of non-restraint practised in our asylums. To this report he has also added several reflections of great interest on the causes which foster the progressive increase of lunatics in asylums.

It is gratifying, although at the same time we must confess somewhat astonishing and amusing, to read the expressions of admiration and wonder in which Dr. Morel couches his opinions on the happy results of the system of management now, with hardly an exception, adopted in the wards of our public asylums. We were scarcely prepared to find that in this respect we had surged so far a-head of our neighbours, or that the labours of Dr. Conolly, either literary or practical, and their results, had been so imperfectly appreciated across the straits. However, Dr. Morel has now proclaimed himself the Gallic champion of the non-restraint system, and on his shield he has engraved the legend "*Restraints and neglect may be considered as synonymous.*"† Assuredly a more gallant knight never laid literary lance in rest.

"Non-restraint," he writes, "is not a factitious thing, a decoy of the understanding, or a piece of charlatanry, as some opponents have not hesitated to aver, but it is wholly a system of treat-

---

* *Le Non-Restraint, ou de l'Abolition des Moyens Coercitifs dans le traitement de la Folie, suivi de considérations sur les causes de la Progression dans les nombre des Aliénés admis dans les Asiles.* Par M. le Dr. Morel, Medicin en Chef de l'Asile de Saint-Yon (Seine Inferieure).

† Conolly: *On the Treatment of the Insane.*

ment. I can affirm in my turn that, my peculiar prejudices having yielded to the evidence of facts, I regard *non-restraint* as the highest expression of that which can be realized for the intellectual, physical, and moral amelioration of the lunatics confided to our care. The reason of this may be readily understood. *Non-restraint* is but the culmination of all the improvements which it has been sought to establish in an asylum prior to giving to the patients that liberty which the English physicians have finished by carrying into effect, and which has made their public establishments for the insane models of order and tranquillity." (p. 37.)

This is no hasty conclusion, for Dr. Morel is careful to tell us that he was not contented to run hastily through our asylums. "I sojourned there," he writes, "I lived the life of the physicians, and I might say of the attendants and patients. No detail, however intimate, of this life escaped me at any instant, day or night. I was able, at any hour and alone, to go and come, to converse with the lunatics, to witness narrowly how they were cared for and treated; I was, in a word, as free in my movements, as little impeded in my observations, as if I had been in my own asylum at Saint-Yon." (p. 20.)

But there were certain preliminary questions to be set at rest before it could be accepted by Dr. Morel that the order and quietude of the wards of English lunatic asylums, particularly as compared with those of the French, were to be attributed chiefly to the system of non-restraint.

Might it not be that the English lunatic differed from the French in being less excited and less excitable; that the general paralytic was less violent and disorderly in his acts; that the epileptic was less furious before and after the paroxysms of his malady; that the idiot was less impulsive and less degraded, not manifesting those periodical outbursts of excitement and mischievousness observed in France? Or could it be that the medical service was better organized; that the English physician had at his command better, more zealous, and more devoted agents; or that the natural disposition of the insane to violence and to irritability was tempered by the greater comfort they enjoyed from the kind of nourishment, of clothing, warmth and ventilation—from, in short, all the elements which concur to soothe the morbid activity of the senses and calm the exacerbations of the nervous system?

The question of race is readily dealt with. The frightful state of our asylums before the introduction of the non-restraint system sufficiently proves that the agitation and fury of the lunatic is less dependent upon the temperament and character of a people than has been sometimes thought. "On the other hand, "the intrinsic nature of a malady such as mental alienation remains always the same." The general paralytic, the idiot, the demented, the imbecile, the epileptic, and the mentally deteriorated of any kind, present in every country the same characteristics; hence this question (if it could be taken into serious consideration) may also be set aside.

We cannot claim any advantage over our neighbours in the medical organization of our asylums, but Dr. Morel believes that the intellectual and moral status of our subordinate agents is higher than theirs. This is doubtless to be accounted for by the superior rate of remuneration given to the attendants in English asylums. The *domestic* arrangements of the wards of our asylums are, it is admitted by our author, in several respects better than those of the French asylums, and it cannot be denied that the maintenance of order and quietude among the insane is in a great degree dependent upon the comforts with which they are surrounded.

In respect to the classification of the insane it would seem that we possess several important advantages. The following observations of Dr. Morel possess somewhat more than a passing interest:—

"It has appeared to me," he writes, "that the English asylums do not serve in so absolute a manner as ours as receptacles for those sad and terrible infirmities and states of degradation of our species that I am about to mention. In the asylums that I visited I found fewer general paralytics, fewer instances of senile dementia, and scarcely any idiots and imbeciles. The number of epileptics was also less. On the other hand, the asylums that I saw in England were exclusively devoted to the indigent; lunatics of the wealthier classes being placed in particular institutions. This fact alone indicates, then, that a selection is exercised among the insane who are secluded in the great English establishments. These institutions do not receive, for example, those degraded natures, instinctively malicious, a singular melange of perversity and insanity, who are sent from our prisons to our asylums, but who in England remain in the places where they were primarily lodged, especially when the individuals have been guilty of murder, incendiarism, robbery, or other crimes. But it must not be concluded from this that the insane received into English asylums are all models of calmness and gentleness, and that, from the nature of their temperament, they are predisposed to live without a strait-jacket.

"From what I have said of the relatively less number, in English asylums, of idiots, imbeciles, the demented from age, and the degenerated of every species which encumber the French asylums, it ought not to be inferred, however, that England in this respect is more favoured than we are. The conclusion could not be sustained in presence of statistical records, or even of the general study which I was enabled to make, although in a very imperfect manner, of the physical and moral perturbing causes existing in the United Kingdom. There, as in France, these causes act with very great intensity, and produce in like manner insanity, as well as all those degradations of human nature which are the consequences of that action, and which, in my *Treatise on Degenerations*, I have designated by the term *unhealthy varieties* (variêtés maladives) *in the human species* . . . .

" . . . . It may be demonstrated that the number of lunatics confined in the asylums of the countries I have mentioned (England and Wales) is far from representing the mass of those who exist in reality. I suspect, although it might be difficult for me to attest this positively, that it is not always either the most troublesome by their turbulence, or by their congenital or acquired infirmities, or the most dangerous by the nature of their acts, who are secluded in the asylums. There exists, in fact, in England an incontestible tendency to establish categories, and not, as with us, to suffer a confusion of so many clashing elements which renders it so difficult to maintain order and discipline in an establishment for the insane, as well as to apply an exclusive system, which sets aside every means of coercion, in ordêr to maintain and reform so many dangerous natures. This method of classification is it preferable to the system of isolation which chiefly exists in our country, and will there not come a period when the imbeciles and idiots and all that which may be called the *caput mortuum* of alienation, will in England, as in France, force the gates of the asylums and still further increase the sad over-crowding which exists in the greater number of those establishments?" (pp. 21-23.)

Admitting that we possess the tendency here attributed to us by Dr. Morel, of separating the unsound of mind into certain categories and providing for these categories apart the one from the other; admitting, also, that this tendency facilitates the adoption of measures calculated to promote order and discipline in an asylum; still, notwithstanding this advantage, and the advantages possessed by the greater degree of domestic comfort in our asylums, as well as by the better class of subordinate agents employed in them, yet there was a wide margin of amelioration to be accounted for, and which was to be attributed to the English system of non-restraint. To this, as we have seen, Dr. Morel does full justice.

Dr. Morel's observations on the absence from our asylums of those classes of persons of unsound mind who form a permanent source of many social mischiefs, and who, indeed, constitute the chief source of those social evils which have their origin in lunacy, are of very grave interest. The opinions of Dr. Morel upon this subject confirm a belief we have long entertained of the inadequacy of our great asylums, and present asylum system, to affect permanently for good the fostering causes of lunacy in the country. Our great ayslums are simply receptacles for the amount of insanity which is thrown prominently up to the surface of society, and their influence as curative institutions is of the very weakest. Strive as we may, our efforts are insufficient to meet even the ordinary requirements of the country for known lunacy alone; and we are compelled, against our own will, to make use of our *workhouses* as lunatic receptacles, notwithstanding that in so doing we know that in several important respects we promote the evil we are seeking to ameliorate. In fact, we go on from year to year dealing in an empirical manner with so much lunacy as is forced upon our attention, but caring not a whit to seek from whence it comes. We act, indeed, somewhat in the fashion of the ingenious peasant, who, knowing that the roof of his cottage is somewhat dilapidated, and that it affords but an imperfect protection from the rain, is content to bring into use every vessel that he may have, good, bad, or indifferent, to catch intrusive streams of water, but never dreams of ascending to the roof and seeking for the inlets of the streams, and so stopping the mischief at its source. It would be folly on our part to waste time in the utterance of regrets that the great and almost sole means we possess of obtaining an accurate account of the status of lunacy in this country, and a firm basis of inquiry into the fostering causes of lunacy among us, should have been, in the past year, again neglected. We allude to the Census. It has been definitively decided that insanity shall have no part in this national inquiry now once more close at hand. This omission we can only explain upon the supposition that neither the Government nor the people are as yet sufficiently awake to the important national and social interests involved in the subject. We had thought that the mere questions concerning the enormous and increasing expenses for the maintenance of lunatics, the insufficiency of the means provided for their care, and the growing difficulties experienced both administratively and pecuniarily to meet even the most immediate requirements, would have been sufficient, apart from any other considerations, to show the importance of ascertaining fully the present and prospective difficulties with which we had to cope. We had thought, indeed, that if all the items of the problem to be solved were known, a solution might be perhaps more satisfactorily and certainly arrived at. We erred, however, in opinion; but our course lays still plain before us—to wit, to seek by all means in our power to arouse a greater interest in, and attention to, not merely the financial evils (so to speak), but also, and above all, to the great moral and social evils arising from lunacy. Lunacy is something more than a mere disease to be looked at from a purely medical point of view. It is a sad but true index of the activity of those causes which most impede the onward progress of civilization, and which form the bane of that progress. It is, moreover, but a more defined state of intellectual and moral deterioration, the lower grades of which are disseminated widely among society. Upon the influence which we can exercise upon these lower grades will, in the end, depend our ultimate hopes of diminishing the amount of lunacy among us.

Dr. Morel's conclusions concerning the increase of lunatics are of great interest:—

"The progressive augmentation of lunatics in our asylums," he writes, "is not indefinite, and a moment will arrive, in the evolution of the causes of deterioration of the human species, when the evil will be modified by its own excess."

And this is the mode in which the evil will work its own cure —a cure, however, which it is not too improbable to surmise would be contemporaneous with the decline of a race or nation. "A great number of individuals, congenitally stricken with alienation or mental imbecility, would disappear from the world in consequence of their slight viability and their want of all power to propagate. A number not less considerable, sad to say, will spare the administration the expense of maintaining them in an asylum by committing suicide." Elsewhere (p. 95) our author writes:—"In France the number of suicides, according to statistical records, amounts to nearly 3000 individuals of both sexes, and (this is an incontestible truth) four-fifths of these unfortunates are lunatics."

The practical conclusions following in order after the foregoing, although intended especially for France, do not fail in interest for us:—

"Notwithstanding the enormous sacrifices which are required of towns and districts in consequence of the admission of a multitude of idiots, imbeciles, or demented persons into our asylums, it is undoubted, setting aside even those humane principles which ought to guide us in such a matter, that the sequestration of these beings is less incommodious or dangerous, and is more in the interest of society, than if they were left at liberty or in charge of their families." Dr. Morel points out that idiots, imbeciles, and the intellectually degraded of every kind, including cretins, are not usually regarded as dangerous. But daily observation gives the lie to this opinion. The erotic instincts and the propensity to vagabondage found among these unfor tunates need alone be cited in illustration.*

* See for examples of the evils arising from erotic instincts and vagabondage of idiots and imbeciles, the *Supplement to the 12th Report of the English Commissioners in Lunacy*, and the *Appendices to the 1st and 2nd Reports of the Scotch Commissioners in Lunacy*, also the *Journal of Psychological Medicine*, vol. xii. pp. 344-347; vol. xiii. pp. 392-394.

"The idea that has often been expressed of placing idiots, imbeciles, and the demented in special establishments would augment the expense of administration, and would, up to a certain point, be prejudicial to science, scattering abroad the elements necessary to favour our studies upon the causes which produce alienation and the degeneration of the human species.

"But, as the study of these causes has already revealed the predominance of forms complicated with paralysis and dementia, and which constitute the states of congenital debility, with production of instincts more or less dangerous, it is evident that the new architectural conditions of our asylums ought, in future, to be in relation with the greater frequency of the degenerated states that I have mentioned.

"Without estimating the economy that these new architectural conditions would lead to, since their character would have relation to the exigencies of a rural life, they would also tend to secure more promptly the different needs of the service, to establish a more decisive line of demarcation between the categories so widely separated by their instincts, their manners, their habitudes, and the dissimilarity resulting from different lesions of the nervous system. They should especially (better than has been hitherto done) preserve patients who are boarders from contact with the indigent. This is with families a subject of incessant complaints, and recriminations and regrets more or less legitimate.

"When it has been universally admitted that the imperfect beings of whom I speak cannot be cared for at home without imposing upon families sacrifices above their strength, and that, on the other hand, it is impossible, without the greatest danger to these kinds of infirm patients and to society, to leave them at liberty, it will be necessary to resolve to place them in asylums to be established in every district." (pp. 99—100.)

We shall leave these conclusions to tell their own story; but there is a lesson to be learned in medical observation from Dr. Morel's report, too good to be passed by unheeded. In a letter addressed to the Prefect of the Seine-Inférieure, Dr. Morel points out the importance of instituting an inquiry, based upon statistics, into the state of lunacy and allied conditions of intellectual and moral deterioration in the department, and he suggests a form of inquiry.

"—The greater or less frequency of insanity and of the diverse degenerations of the human species in this or that country are always in relation with the greater or less frequency of the perturbing causes of physical and moral health of the inhabitants of the country. The programme to be followed, in order to study well the consequences of these causes, may be summed up in the following questions :—

"What is the morality of the inhabitants within a certain boundary? To answer this question it will be necessary to know the number of illegitimate children, and those of crimes against the person and against property. It will be needful also to estimate the suicides, the extension of prostitution, the amount of natural and accidental deaths, &c.

"What are the food and hygiène of the inhabitants? What are the predominant maladies within this or that boundary? What influence is exercised by the industry, the manner of living, the nature of the soil, and the kind of its culture upon the habits, the temperament, the morality and the physical health of the individuals? What are the most frequent causes of exemption from conscription?

"3. What is the state of primary instruction in each of the communes? What, in the departments, are the most ordinary causes of intellectual excitability and of moral emotions?

"4. What is especially the proportion of drunkenness, and what is the quantity of alcoholic drinks consumed? What effect has this deadly habit of drinking upon the sterility of woman, upon the viability of infants, upon vagabondage, upon criminal precocity, upon congenital idiocy and imbecility, &c.?"

It will be seen at a glance that many of the more important of these data with us are furnished by the Registrar-General's Reports, the judicial statistics published by the Home Office, the returns of the Poor-Law Board and the Board of Trade; but much would still be wanting. Until, however, we have a better knowledge than we now possess of the status of insanity among us no systematic inquiry in this country or in any district of it (as a rule) would be practicable.

Before closing Dr. Morel's report we would note that he has thought it necessary incidentally to defend himself from an imputation of elevating the merits of Dr. Conolly at the expense of Pinel. In defending himself from this charge, he writes, "If the idea of reform *apropos* of the insane appertains incontestably to Pinel, it does not follow that those who have carried into effect a method so salutary as that of *non-restraint* have not also introduced a novelty in the asylums over which they had control. The idea of Pinel contained in germ everything that has been effected since; but it was not sufficient to break the chains of the insane; it was requisite to attain a more complete realization, a more general application of this humanizing idea. Now, it seems to me that the English physicians have taken this course, although I do not overlook certain exaggerations which I am far from approving, but which are inseparable from the excitement of working out any idea of reform." (p. 39.)

